# The correlation of SUVmax with pathological characteristics of primary tumor and the value of Tumor/ Lymph node SUVmax ratio for predicting metastasis to lymph nodes in resected NSCLC patients

**DOI:** 10.1186/1749-8090-8-63

**Published:** 2013-04-04

**Authors:** Deniz Koksal, Funda Demirag, Hulya Bayiz, Ozlem Ozmen, Ebru Tatci, Bahadir Berktas, Koray Aydoğdu, Erdal Yekeler

**Affiliations:** 1 Chest Diseases Clinic, Ataturk Chest Diseases and Chest Surgery Education and Research Hospital, Ankara, Turkey; 2 Pathology Department, Ataturk Chest Diseases and Chest Surgery Education and Research Hospital, Ankara, Turkey; 3 Nuclear Medicine Department, Ataturk Chest Diseases and Chest Surgery Education and Research Hospital, Ankara, Turkey; 4 Chest Surgery Clinic, Ataturk Chest Diseases and Chest Surgery Education and Research Hospital, Ankara, Turkey

## Abstract

**Background:**

We aimed to investigate the correlation of maximum standardized uptake value (SUVmax) with pathological characteristics of primary tumor and to determine a Tumor/ Lymph node (T/LN) SUVmax ratio predicting metastasis to lymph nodes in NSCLC patients.

**Methods:**

Eighty-one NSCLC patients who had PET/CT examination at initial staging and subsequently underwent surgical resection were retrospectively evaluated. There were 100 PET/CT positive mediastinal or hilar lymph node stations. Pathological characteristics of the tumor such as largest tumor diameter, tumor histology, differentiation, number of mitosis, degree of stromal inflammation, necrosis; etiology of PET/CT positive lymph node stations; SUVmax of primary tumor and positive lymph node stations were recorded. A T/LN SUVmax ratio was calculated for each lymph node station.

**Results:**

SUVmax of the primary tumor was positively correlated with the largest tumor diameter (p = 0.001, r = 0.374), number of mitosis (p < 0.001, r = 0.405), and postoperative pathological stage (p = 0.007, r = 0.298). Patients with squamous cell carcinoma had a statistically significant higher mean SUVmax, number of mitosis and advanced N stages compared to adenocarcinoma. The etiology of 100 PET/CT positive lymph node stations were metastasis in 14, anthracosis in 40, reactive in 39, granulomatous in 4, and silicosis in 3 patients. A T/LN SUVmax ratio of 5 or lower was suggestive for a malignant lymph node with a sensitivity of 92.8% and specificity of 47%.

**Conclusions:**

SUVmax of a primary tumor is related to certain pathological characteristics, such as largest diameter, histology, and number of mitosis. A T/LN SUVmax ratio lower than 5 predicts the metastasis to lymph nodes with a high sensitivity.

## Background

Non-small cell lung cancer (NSCLC) is a heterogeneous group of carcinomas with different biological behaviors and prognoses. Histological classification and staging are critical in constituting a treatment strategy and predicting prognosis for NSCLC [1]. [^18^ F]-2-fluoro-deoxy-D-glucose (FDG)-positron emission tomography (PET) is a metabolic imaging technique which has become an essential tool for staging of NSCLC patients. The integration of PET with computed tomography (PET/CT) provides an accurate anatomic localization and improved staging especially for mediastinal lymph nodes and occult distant metastases [2,3]. It also provides prognostic information, monitors response to therapy and can be used to follow up patients after treatment [4]. The rationale for using FDG-PET in oncology is its ability to measure increased glucose metabolism of tumor cells. Elevated FDG uptake suggests that the lesions or tissues harbor tumor cells. The maximum standardized uptake value (SUVmax) greater than 2.5 is often used as a cut off value for malignancy. However it has been shown that there is a significant number of false positivity (due to inflammatory diseases) and false negativity (due to low-grade malignancies) in the evaluation of primary tumor [5]. The major reasons of false negative and false positive lymph nodes are microscopic metastasis beyond the spatial resolution of PET/CT and lymph node involvement by underlying inflammatory processes such as immune reaction due to the presence of lung tumor, obstructive pneumonia, anthracosis or granulomatous inflammation [6-8].

It is often assumed that FDG uptake is primarily within the malignant tumor cells, and SUVmax is a well known measure indicating the aggressiveness of tumor [9,10]. But other cellular components of such as normal parenchymal cells, atypical cells, inflammatory cells, fibroblasts, or hematopoietic progenitor cells may also uptake FDG. To the best of our knowledge, there is only one study investigating the correlation between SUVmax and specific cellular components of the tumor conducted in patients with resected stage 1 NSCLC. In that study the authors found that the cellular composition of the tumor was highly variable and there wasn’t any correlation between a specific tumor cellular component and FDG activity [11].

SUVmax of the primary tumor is a risk factor for occult mediastinal metastasis in clinical stage 1 NSCLC patients [12-14]. The likelihood of lymph node metastasis increases with the increase of tumor SUVmax [6,15]. SUVmax of lymph node is also important for predicting metastasis, but false positivity is an important challenge [6,7]. For this reason some authors claimed to use a higher SUVmax value instead of the traditional value of 2.5 in order to increase the accuracy for presence of metastasis [7].

In this study we aimed to investigate the correlation of tumor SUVmax value with largest tumor diameter, tumor histology, differentiation, number of mitosis, degree of stromal inflammation and necrosis, and to determine whether a T/LN SUVmax ratio can predict the presence of metastasis in mediastinal or hilar lymph nodes in NSCLC patients.

## Methods

### Patients

Eighty-one chemotherapy and/or radiotherapy naive NSCLC patients with a PET/CT examination at the time of initial staging who subsequently underwent surgical resection were retrospectively evaluated. Among them 77 were male and 4 were female with a mean age of 59.8 ± 8 years (range: 38-74). Fifty-five patients underwent lobectomy, 25 patients pneumonectomy and 1 patient wedge resection with complete ipsilateral hilar and mediastinal lymph node dissection. Pathological stage was determined according to 7th edition of TNM staging system [1]. Demographic data of the patients, histopathological diagnosis, T and N stages are presented in Table 1. This retrospective study was approved by Institutional Review Board Committee.

**Table 1:** Patient characteristics

**Characteristic**	**Number of patients (%)**
Male/Female	77 male/4 female
Mean age (years)	59.8 ± 8 (range: 38-74)
**Type of surgery**	
Lobectomy + LND	55 (67.9%)
Pneumonectomy + LND	25 (30.9%)
Wedge resection + LND	1 (1.2%)
**Tumor histology**	
Adenocarcinoma	32 (39.5%)
Squamous cell carcinoma	43 (53.1%)
Adenosquamous carcinoma	4 (4.9%)
Pleomorphic carcinoma	2 (2.5%)
**Adenocarcinoma classification**	
Acinar predominant	16 (50%)
Solid predominant	9 (28.1%)
Papillary predominant	3 (9.4%)
Lepidic predominant	2 (6.2%)
Minimally invasive mucinous	2 (6.2%)
**Differentiation of squamous cell carcinomas**	
Well	26 (60.5%)
Moderate	15 (34.9%)
Poor	2 (4.6%)
**T Stage**	
1a	1 (1.2%)
1b	10 (12.3%)
2a	28 (34.6%)
2b	15 (18.6%)
3	26 (32.1%)
4	1 (1.2%)
**N Stage**	
0	47 (58%)
1	22 (27.2%)
2	12 (14.8%)
***Stage***	
*IA*	8 (9.9%)
*IB*	17 (21%)
*IIA*	17 (21%)
*IIB*	21 (25.9%)
*IIIA*	18 (22.2%)

### PET/CT imaging

PET/CT was carried out with an integrated PET/CT scanner *(Siemens, Biograph-6- True Point)* within the 30 days before the surgery in all of the patients. Patients were instructed to fast for at least 6 hours before the examination. After confirmation of a normal peripheral blood glucose level (<180 mg/dL), the patients received an intravenous injection of 145 μCi/kg (maximum 200 μCi) of FDG and rested for 60 minutes before the scan. Images were obtained from the base of skull to mid-thigh level. The SUVmax of the primary tumors and each suspicious lymph node stations were determined automatically by the software after delination of the region of interest on attenuation-corrected PET/CT images.

All PET/CT scans were reevaluated. SUVmax of the primary tumors and dissected mediastinal and hilar lymph node stations were noted. Lymph node stations were considered as positive if there was a FDG uptake higher than the surrounding mediastinal blood pool. The SUVmax value of positive lymph node stations were ranging between 1.57 and 24.75 (median: 3.15, mean ± SD: 3.57 ± 2.45). Tumor/ lymph node SUVmax (T/LN SUVmax = tumor SUVmax/ lymph node station SUVmax) ratio was calculated for each lymph node station.

### Pathological evaluation

All prepared hematoxylin-eosin stained slides of each resected tumor and lymph nodes were re-evaluated by an experienced pulmonary pathologist blinded to PET/CT results and other clinical data of the patients. The histological subtype and the largest primary tumor diameter were noted. Invasive adenocarcinomas (AC) were classified according to the new adenocarcinoma classification using the predominant histology in a tumor, as lepidic predominant, acinar predominant, papillary predominant, micropapillary predominant, and solid predominant with mucin production [16]. The differentiation of squamous cell carcinomas (SCC) was noted. All of the slides of the tumor were evaluated for the number of mitosis, presence of necrosis, and stromal inflammation. Number of mitosis was counted in the 10 consecutive high power field without necrosis. Necrosis was graded as “0” if there wasn’t any necrosis in all the slides, “1” if there was necrosis in only one slide, “2” if there was necrosis in two slides, and “3” if there was necrosis in more than two slides. Stromal inflammation was graded semi-quantitatively as mild, moderate, and severe.

Three hundred and thirty-four lymph node (mediastinal and hilar) stations from 81 patients were reevaluated for the presence of metastasis. The number of lymph nodes in each station was not noted. Interlobar and intrapulmonary lymph nodes were excluded. PET positive lymph node stations (n = 100) were investigated for the presence of reactive lymphoid proliferation, anthracosis, granulomatous reactions, and silicotic nodules [17].

### Statistical analysis

SPSS for windows release 19.0 package program was used to carry out the statistical analysis and construct figures. The descriptive analysis was expressed in terms of frequency, median, mean and standart deviation. Comparisons of ordinal parameters between different groups were performed by Chi-square test. Comparisons of continuous parameters between different groups were performed by nonparametric Mann Whitney-U and Kruskal-Wallis test. Spearman’s rank correlation was performed to analyze the correlation between variables, such as SUVmax, largest tumor diameter, number of mitosis, degree of necrosis and stromal inflammation. Receiver operating characteristics (ROC) curve analysis was generated that maximized the sensitivity and the specificity and thus the accuracy for assessing a cut off value for T/LN SUVmax ratio. A p value less than 0.05 was considered to be statistically significant.

## Results

### Evaluation of the primary tumor

The pathological characteristics and mean SUVmax of the tumors are shown in Table 2. Mean diameter of the tumors were 4.86 ± 1.93 cm (range: 1.5-10). Mean SUVmax of the tumors were 14.23 ± 7.23 (range: 2.23-38.76). Mean number of mitosis, median degree of necrosis and stromal inflammation were 27.9 ± 17.2 (range: 2-80), 1 (range: 0-3) and 2 (range: 1-3), respectively.

**Table 2:** Tumor characteristics

**Characteristic**	
Mean tumor diameter	4.86 ± 1.93 cm (range: 1.5-10)
Mean tumor SUVmax	14.23 ± 7.23 (range: 2.23 ± 38.76)
Mean number of mitosis	24.91 ± 17.18 (range: 2-80), Median: 21
**Grade of necrosis**	
0	27 (33.3%)
1	17 (21%)
2	12 (14.8%)
3	25 (30.9)
Median grade of necrosis	1 (range: 0-3)
**Grade of stromal inflammation**	
1	32 (39.5%)
2	37 (45.7%)
3	12 (14.8%)
Median grade of stromal inflammation	2 (range: 1-3)

**Figure 1:**
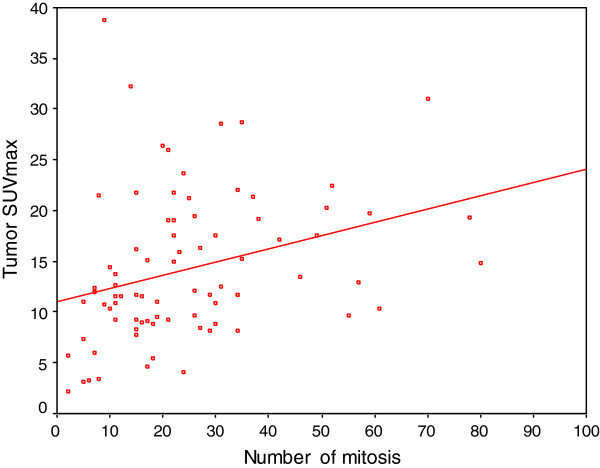
There was a positive correlation between tumor SUVmax and number of mitosis (p < 0.001, r = 0.405).

SUVmax of the tumors were positively correlated with the number of mitosis (p < 0.001, r = 0.405) (Figure 1). There was no correlation between the tumor SUVmax and degree of stromal inflammation (p = 0.381) or degree of necrosis (p = 0.834). SUVmax was positively correlated with the tumor diameter (p = 0.001, r = 0.374). The diameter of the tumors were positively correlated with the degree of necrosis (p < 0.001, r = 0.521).

The pathological stage of the disease was positively correlated with the tumor diameter (p < 0.001, r = 0.503), SUVmax (p = 0.007, r = 0.298), number of mitosis (p = 0.038, r = 0.231) and the degree of necrosis (p = 0.002, r = 0.335). T stage was positively correlated with the tumor diameter (p < 0.001, r = 0.725), SUVmax (p = 0.005, r = 0.309), number of mitosis (p = 0.048, r = 0.220) and the degree of necrosis (p < 0.001, r = 0.412). There was no correlation between the N stage and tumor SUVmax (p = 0.630).

Tumor characteristics of AC and SCC were compared in Table 3. Two groups had a similar T stage (p = 0.25). N stages were advanced in SCC (p = 0.017). Tumor SUVmax (p = 0.042) and number of mitosis (p < 0.001) were significantly higher in SCC. Tumor diameter was positively correlated with the SUVmax of the tumor both in the AC (p = 0.003, r = 0.508) and SCC patients (p = 0.002, r = 0.453). We also compared the tumor SUVmax/ tumor diameter ratios in order to eliminate the effect of tumor diameter on SUVmax and showed that SCC had a significantly higher ratio (p = 0.006).

**Table 3:** The comparison of tumor characteristics of adenocarcinomas and squamous cell carcinomas

	**Adenocarcinoma (n = 32)**	**Squamous cell Ca (n = 43)**	**p**
Tumor diameter (cm) (mean ± SD)	4.9 ± 2.2	4.7 ± 1.6	0.93
Tumor SUVmax (mean ± SD)	12.6 ± 8.3	15 ± 5.9	0.042*
Number of mitosis (mean ± SD)	16.12 ± 12.03	31.5 ± 18.0	<0.001*
Necrosis (median)	1	2	0.24
Stromal inflammation (median)	2	2	0.08
Tumor SUVmax/Tumor diameterÞ (mean ± SD)	2.64 ± 1.52	3.50 ± 1.71	0.006*

*Statistically significant.

Þ In order to eliminate the effect of tumor diameter on the SUVmax values, we compare the tumor SUVmax value/ tumor diameter ratios.

According to the new AC classification, the study group consists of 16 acinary, 9 solid, 3 papillary, 2 lepidic, and 2 mucinous AC patients (Table 1). We only compared acinary AC with solid AC because of low number of cases in the last three groups (Table 4). Mean tumor diameter (p = 0.014), number of mitosis (p = 0.018) and grade of necrosis (p = 0.001) were significantly higher in solid AC. Although the tumor SUVmax was higher in solid AC, it did not reach a statistical significance. We also compared the tumor SUVmax/ tumor diameter ratios and found no difference between acinary and solid AC (p = 0.73).

**Table 4:** The comparison of tumor characteristics of acinary and solid adenocarcinomas

	**Acinary (n = 16)**	**Solid (n = 9)**	**p**
Tumor diameter (cm) (mean ± SD)	4.7 ± 1.8	6.8 ± 2.1	0.014*
Tumor SUVmax (mean ± SD)	12.9 ± 7.5	16.7 ± 8.1	0.21
Number of mitosis (mean ± SD)	12.8 ± 7.1	26.7 ± 15.4	0.018*
Necrosis (median)	1	2	0.002*
Stromal inflammation (median)	2	2	0.48
Tumor SUVmax/Tumor diameter¥ (mean ± SD)	2.90 ± 1.49	2.63 ± 1.44	0.73

*Statistically significant.

¥ In order to eliminate the effect of tumor diameter on the SUVmax values, we compare the tumor SUVmax value/ tumor diameter ratios.

The differentiation of SCC is shown in Table 1. There was no correlation between the differentiation of the tumor and SUVmax (p = 0.61), number of mitosis (p = 0.9), degree of necrosis (p = 0.2). Stromal inflammation was higher in poorly differentiated SCC (p = 0.03, r = 0.31).

### Evaluation of the lymph nodes

A total of 334 mediastinal and hilar lymph node stations were evaluated. Among them, there were 100 lymph node stations showing higher FDG uptake than the surrounding mediastinal blood pool (SUVmax range: 1.57-24.75, median: 3.15, mean ± SD: 3.57 ± 2.45) on PET/CT. Fourteen (14%) of them were due to metastasis, 39 (39%) reactive, 40 (40%) anthracosis, 4 (4%) granulomatosis and 3 (3%) silicosis. All of the metastatic lymph node stations (10 mediastinal, 4 hilar) had a SUVmax ≥2.5. Twenty-four patients (29.6%) had metastatic interlobar or intrapulmonary lymph nodes that could not be identified on PET/CT because of close relationship with the primary tumor.

PET/CT evaluation and the presence of metastasis in the mediastinal and hilar lymph node stations are shown in Table 5. The sensitivity, specificity, and accuracy of PET/CT for the staging of mediastinal and hilar lymph nodes were 63.6%, 72.4%, and 71.8% respectively. False positive and false negative lymph node ratios were 86% and 3.4%, respectively.

**Table 5:** The PET/CT evaluation and presence of metastasis in mediastinal and hilar lymph node stations

**PET/CT**	**Metastasis (+)**	**Metastasis (-)**	**Total**
Positive§	14	86	100
Negative	8	226	234
**Total**	**22**	**312**	**334**

§A quantifiable SUVmax value is considered as positive.

-The sensitivity of PET/CT: 63.6%, the specificity of PET/CT: 72.4%.

-False positive lymph node ratio 86%, false negative lymph node ratio 3.4%.

Metastatic and non-metastatic lymph node stations are compared in Table 6. Mean lymph node SUVmax was higher in the metastatic lymph nodes, but did not reach statistical significance (p = 0.06). T/LN SUVmax ratios were significantly lower in the metastatic lymph nodes (p = 0.01). The comparison of metastatic, anthracotic and reactive lymph nodes are seen in Table 7. Mean LN SUVmax (p = 0.04) and T/LN SUVmax ratios (p = 0.02) were significantly different among the groups. Mean LN SUVmax (p = 0.12) and T/LN SUVmax ratios (p = 0.29) of the anthracotic and reactive lymph nodes were similar. Metastatic lymph nodes had a significantly higher mean LN SUVmax (p = 0.018) and significantly lower T/LN SUVmax ratios (p = 0.006) compared to anthracotic LN. Mean LN SUVmax was higher in the metastatic lymph nodes compared to reactives but the difference did not reach to statistical significance (p = 0.15). T/LN SUVmax ratios were significantly lower (p = 0.034) in the metastatic lymph nodes compared to reactive LN.

**Table 6:** The comparision of metastatic and non-metastatic lymph nodes

	**Metastatic (n = 14)**	**Non-metastatic (n = 86)**	**p**
Tumor SUVmax	15.26 ± 6.02	16.5 ± 6.83	0.49
Lymph node SUVmax	5.35 ± 5.72	3.28 ± 1.19	0.06
T/LN SUVmax	3.73 ± 1.49	5.66 ± 3.32	0.01*

*Statistically significant.

**Table 7:** The comparision of metastatic, antracotic and reactive lymph nodes

	**Metastatic (n = 14)**	**Antracotic (n = 40)**	**Reactive (n = 39)**	**p**
Tumor SUVmax	15.26 ± 6.02	16.28 ± 6.95	17.0 ± 7.24	0.78
Lymph node SUVmax	5.35 ± 5.72	2.98 ± 0.86	3.36 ± 1.09	0.04*
T/LN SUVmax	3.73 ± 1.49	5.70 ± 2.79	5.38 ± 2.8	0.02*

*Statistically significant.

-Tumor SUVmax, Lymph node SUVmax and T/LN SUVmax ratios were similar in antracotic and reactive lymph nodes (p > 0.05).

**Figure 2:**
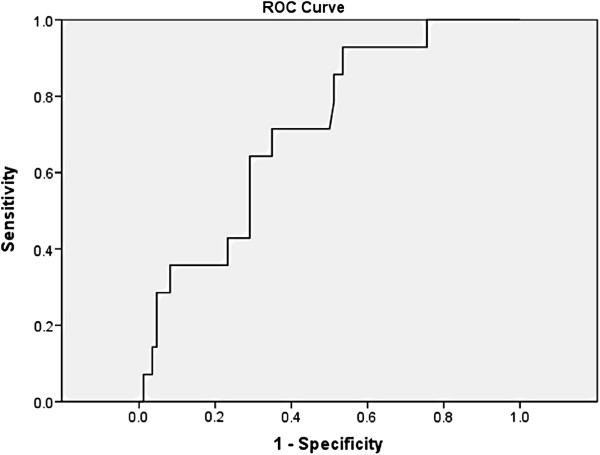
**The receiver operating characteristics (ROC) curve for the optimal cut off value for tumor/lymph node SUVmax ratios.** Area under the curve: 0.716; 95% confidence interval: 0.584 to 0.847; p = 0.01. A T/LN SUVmax ratio of 5 or lower suggest a lymph node to be malignant with a sensitivity of 92.8% and specificity of 47%.

A T/LN SUVmax ratio of 5 or lower was suggestive for a malignant lymph node with a sensitivity of 92.8% and a specificity of 47% (p = 0.01) (Figure 2).

## Discussion

The present study revealed that SUVmax of the primary tumor is positively correlated with the largest tumor diameter, number of mitosis, and pathological stage of the disease. Mean SUVmax and number of mitosis were higher in SCC compared to AC. N stages were advanced in SCC. Reactive hyperplasia and anthracosis were the major etiologies for positive lymph nodes on PET/CT. A T/LN SUVmax ratio of 5 or lower was suggestive for a malignant lymph node with high sensitivity (92.8%) but low specificity (47%).

FDG activity in other words SUVmax measured on PET/CT is a semiquantitative value that indicates the degree of glucose uptake in a lesion. The exact mechanism of FDG uptake remains unknown. Most lung cancers accumulate FDG preferentially that SUVmax may vary widely [11,18]. SUVmax of a tumor is a product of several fundamental factors including glucose metabolism and the type/number of cells present in the tumor. Higher SUVmax may be a result of either higher mitotic activity of tumor cells or higher number of inflammatory cells (lymphocytes, macrophages) that competitively uptake FDG-glucose. Alternatively, there may be a large number of tumor cells with low metabolic activity or a low number of tumor cells with high metabolic activity [11]. In the present study, we found a positive correlation between tumor SUVmax and number of mitosis supporting the role of higher mitotic activity in the mechanism of FDG uptake. There was no correlation between tumor SUVmax and the degree of stromal inflammation.

The tumor size and the presence of necrosis are other factors that affect the SUVmax of a tumor. Previous studies demonstrated the positive correlation between tumor diameter and SUVmax [19-22]. The increase in the tumor diameter was also correlated with more glucose transporter-1 (Glut-1) expression on the surface of tumor cells, leading to increased FDG uptake [23]. In this study, the largest tumor diameter was positively correlated with SUVmax in AC, SCC and the whole study group. As expected, larger tumors were more necrotic. Contrary to the conventional wisdom that tumor necrosis dilutes standart uptake value, more necrosis was not associated with lower SUVmax. There was no correlation between SUVmax and the degree of necrosis. This condition can be explained by the fact that SUVmax is calculated from the highest FDG uptake regions of the tumor.

Consistent with the previous studies [6,19,21,22,24], this study revealed that SCC had higher SUVmax values compared to AC. This can be explained in two ways. First, SCCs are rapidly growing tumors with shorter doubling times, thus leading to higher levels of glucose metabolism [18]. Second, the expression of Glut-1 is higher in SCCs. The localization of Glut-1 is also important in the uptake process of FDG. While Glut-1 is mainly located in cytoplasm of tumor cells in ACs, it is mainly located on cell membranes of tumor cells in SCCs. The cell membrane localization is more important in FDG uptake process [25,26].

Recently, a study investigating Glut-1 expression and FDG uptake in histological subtypes of pulmonary AC documented that solid predominant ACs had a significantly higher Glut-1 expressions and SUVmax than those with other predominant histology [27]. In the present study, despite the fact that there are limited number of cases in AC subgroups, we compared solid predominant ACs (n = 9) with acinary predominant ACs (n = 16 cases) and found that the largest tumor diameters, number of mitosis, and the degree of necrosis were higher in solid ACs. Mean tumor SUVmax was also higher in solid predominant ACs but the difference did not reach statistical significance. All these findings supported the aggressive behavior of solid predominant ACs.

The relationship between tumor SUVmax and differentiation is conflicting in the literature. Some studies showed that tumor SUVmax was higher in poorly differentiated NSCLCs [22,28]. Another study found no correlation between SUVmax and the degree of differentiation in SCCs [19]. In the present study, there was no relation between tumor SUVmax and differentiation in SCCs. But the degree of stromal inflammation was higher in poorly differentiated tumors.

Higher SUVmax of the primary tumor is found to be a strong predictor of lymphtic vessel invasion and lymph node metastasis in studies consisting resected early stage NSCLC patients [21,29]. In a study conducted on a small group of resected lung cancer patients, the authors investigated the correlation of SUVmax with the likelihood of lymph node metastasis and reported that, when the SUVmax of the primary tumor was greater than 12, the probability of lymph node metastasis was high, reaching 70%, irrespective of the degree of FDG activity in the lymph node stations [6]. In the present study, tumor SUVmax was correlated with pathological T stage, disease stage, but not with N stage. Mean tumor SUVmax was not different in patients with and without metastatic lymph nodes (Table 6). Contrary to the current literature reporting AC histology as a risk factor for occult N2 lymph node metastasis [12,13], in this study N stages were advanced in SCCs that have higher SUVmax compared to ACs.

PET/CT is now regarded as the most accurate imaging modality in N-staging of lung cancer. However there are a significant number of false positivity and false negativity. The major reason for false negativity is microscopic metastasis beyond the spatial resolution of PET/CT. The major reasons for false positivity are lymph node involvement by underlying inflammatory disease (tuberculosis, histoplasmosis), lymphadenopathies secondary to obstructive pneumonia, immune reaction due to the presence of lung tumor, antracosis, and silicotic nodules [6,8]. The major cause of false positivity may vary from region to region. In a study from Alabama, histoplasmosis infection was the most common cause of false positivity [7]. Silicosis has been found to be a cause of false positivity in a study from Germany [30]. In the present study, there were antracosis in 40% of the PET/CT positive lymph nodes probably due to the intensive indoor air pollution or biomass exposure. Granulomatous inflammation (4%) and silicosis (3%) was low.

In the present study, distinctively from the literature, we considered a lymph node station as positive if there was a FDG uptake higher than the surrounding mediastinal tissue, with regard to the idea that a tumor with low FDG activity might have a metastatic lymph node with low FDG activity. Thus we set a low threshold (any FDG uptake higher than the surrounding mediastinal blood pool) in order to avoid false negative lymph nodes and accept a higher incidence of false positives. All of the metastatic lymph nodes had a SUVmax higher than 2.5. The sensitivity (63.6%) and specificity (72.4%) of PET/CT was lower than the current literature due to the lower threshold we choose for PET/CT positivity.

In the present study we hypothesized that a T/LN SUVmax ratio could be a predictor of lymph node metastasis. This hypothesis was constituted based on the clinical observation that a cut off value of 2.5 for the prediction of metastasis is too low in countries where inflamatory reactions are more prevelant. Besides in clinical practice we realized that some tumors with low SUVmax values have metastatic lymph nodes after resection despite lower SUVmax values than 2.5 on PET/CT obtained before surgery. The present study favors this hypothesis. As demonstrated in Table 6, while mean SUVmax values were not different between metastatic and non metastatic lymph nodes, T/LN ratios were significantly lower in metastatic ones. T/LN SUVmax ratios were similar in anthracotic and reactive lymph nodes. A T/LN SUVmax ratio of 5 or lower was suggestive for a malignant lymph node with high sensitivity but low specificity. Since this study is performed in a population where the prevalance of inflammatory disease is high, T/LN SUVmax ratios may show variations in different patient populations.

In the literature *Cerfolio et al* also determined a ratio of LN/T SUVmax as a universal predictor of lymph node metastasis in order to eliminate the variation of SUVmax among different PET scanners, and documented that when the ratio is 0.56 or greater, there is a 94% chance that the node is malignant [31].

There are some limitations of the present study. First it is a retrospective study with limited number of patients. The study population consists of patients who underwent surgery only that may cause a selection bias. Since all cases were early lung cancers who are candidates for curative surgery, the number of metastatic lymph nodes was low. Prospective studies including more lymph node stations are needed to examine the validation of a T/LN SUVmax ratio for prediction of metastasis.

## Conclusions

In conclusion, SUVmax of a primary tumor is related to certain pathological characteristics, such as largest diameter, histology, and number of mitosis. A T/LN SUVmax ratio lower than 5 predicts the metastasis to lymph nodes with a high sensitivity.

## Abbreviations

SUVmax: Maximum standardized uptake value; PET/CT: Positron emission tomography with computed tomography; T/LN: Tumor/ Lymph node; FDG: Fluoro-deoxy glucose; NSCLC: Non small cell lung cancer; AC: Adenocarcinoma; SCC: Squamous cell carcinoma.

## Competing interests

All authors disclose that there is not any actual or potential conflict of interest including any financial, personal or other relationships with other people or organizations that could inappropriately influence (bias) their work.

## Authors’ contributions

DK designed the study, collected the data, interpret the results and draft the article. FD carried out the pathological evaluation. HB conceived of the study, and participated in its design and coordination and helped to draft the manuscript. OO carried out the interpretation of PET/CT imagings. ET carried out the interpretation of PET/CT imagings. BB participated in the design of the study and performed the statistical analysis. KA participated in the design of the study and coordination. EY participated in the design of the study and coordination. All authors read and approved the final manuscript.
